# Mitral valve regurgitation and endocarditis triggered by a needle

**DOI:** 10.1186/s12872-021-02109-z

**Published:** 2021-06-13

**Authors:** Chuanzhen Liu, Yan Liu, Jianhua Li, Guangqing Cao

**Affiliations:** 1 Department of Cardiovascular Surgery, Qilu Hospital of Shandong University, Wenhua Xi Road 107#, Jinan, 250012 Shandong Province People’s Republic of China; 2 Pantheum Biotechnology Co., Ltd, Jinan, Shandong Province People’s Republic of China; 3 Department of Cardiology, Qilu Hospital of Shandong University, Wenhua Xi Road 107#, Jinan, 250012 Shandong Province People’s Republic of China

**Keywords:** Endocarditis, Heart failure, Needle, Edge to edge, Case report

## Abstract

**Background:**

Cardiac foreign bodies are extremely rare in clinical patients, especially when foreign bodies damage the internal structure of the heart coincidentally after they penetrate the heart.

**Case presentation:**

Here, we report the case of a two-year-old girl whose heart was penetrated by a needle, which triggered mitral valve regurgitation and endocarditis. After a comprehensive inspection, accurate judgment and surgical preparation, we removed the needle and repaired her mitral valve. Fortunately, she recovered postoperatively.

**Conclusion:**

From this case, we can know that when cardiac foreign bodies are suspected, ultrasound is an important inspection method. Moreover, the approaches for handling each such case are different depending on the associated injuries.

**Supplementary Information:**

The online version contains supplementary material available at 10.1186/s12872-021-02109-z.

## Background

Cardiac foreign bodies are extremely rare in clinical patients [1], and metallic foreign bodies are most reported [2]. They mostly occur in the right ventricle, and this may be related to the anatomical position of the right ventricle. These foreign bodies mostly enter from the left anterior chest close to the sternal stem. Some foreign bodies can also flow into the right heart cavity from the peripheral vein [3]. However, most patients have no typical symptoms and signs.

Because the heart is an organ of continuous movement, trauma resulting from metal foreign bodies remaining in the heart can not only cause hemorrhage and shock but also cause pericardial effusion and even pericardial tamponade, resulting in unpredictable serious consequences, and therefore should be highly regarded. The foreign bodies can sometimes be directly removed by minimally invasive surgery [4]. However, if a foreign body destroys the valves and subvalvular structure of the heart, it can cause heart failure and endocarditis. Therefore, it needs to be paid high attention to and should be treated with thoracotomy under cardiopulmonary bypass. Herein, we present the case of a two-year-old girl with mitral valve regurgitation and endocarditis triggered by a needle.

## Case presentation

**Fig. 1:**
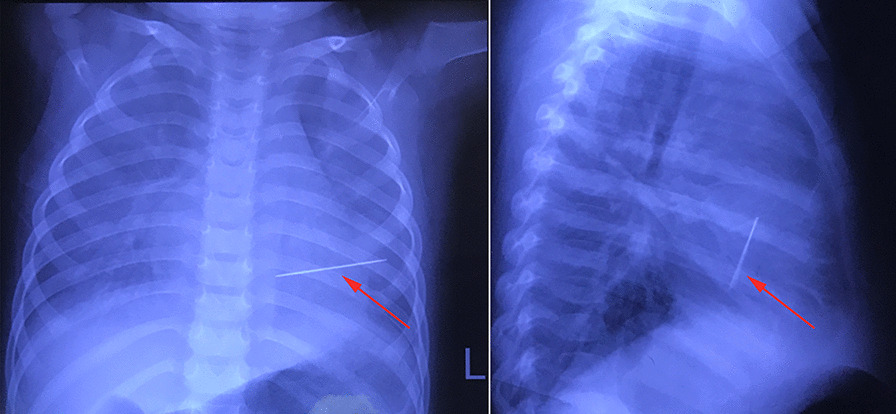
Chest X-ray. Arrows indicated the needle

**Fig. 2:**
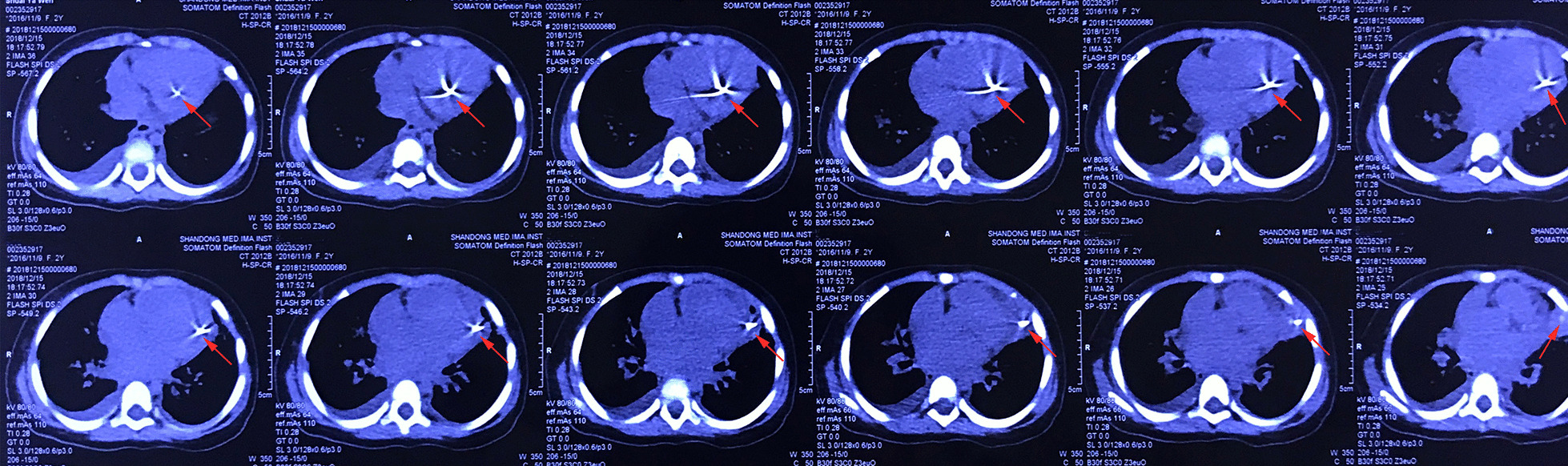
High-quality thoracic CT. Arrows indicated the entry, exit and extension of the needle through the heart

**Fig. 3:**
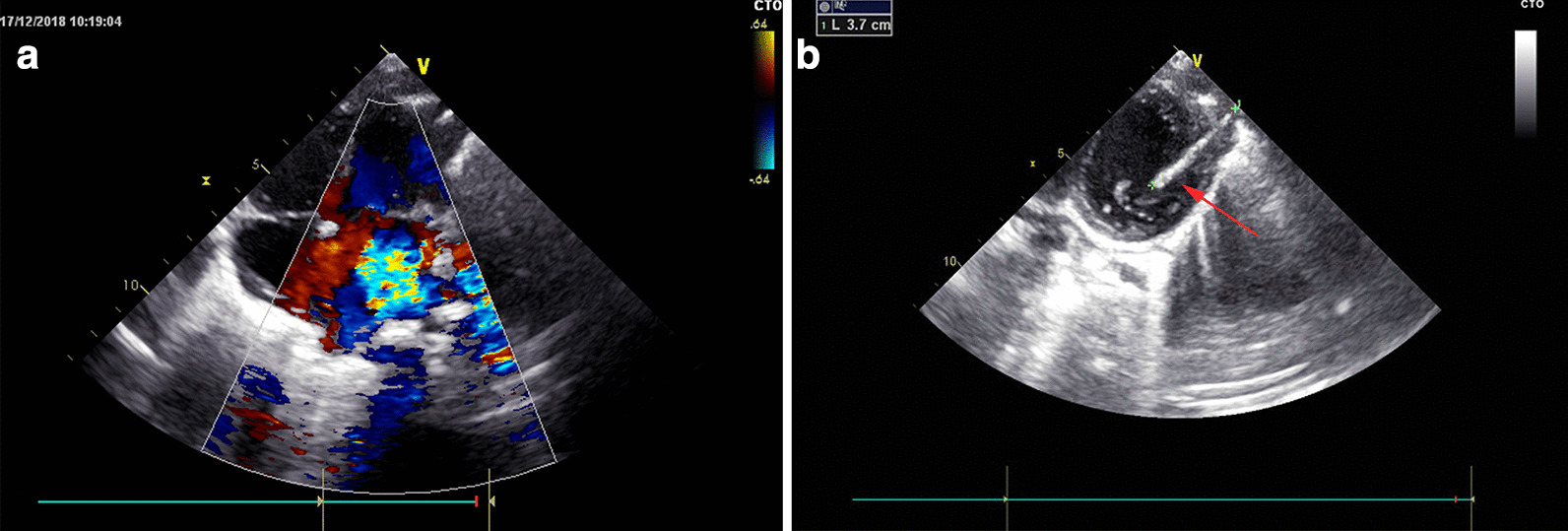
Echocardiography. **A** Echocardiography showed severe mitral valve regurgitation. **B** Echocardiography showed the needle in the heart cavity. The arrow indicated the needle

**Fig. 4:**
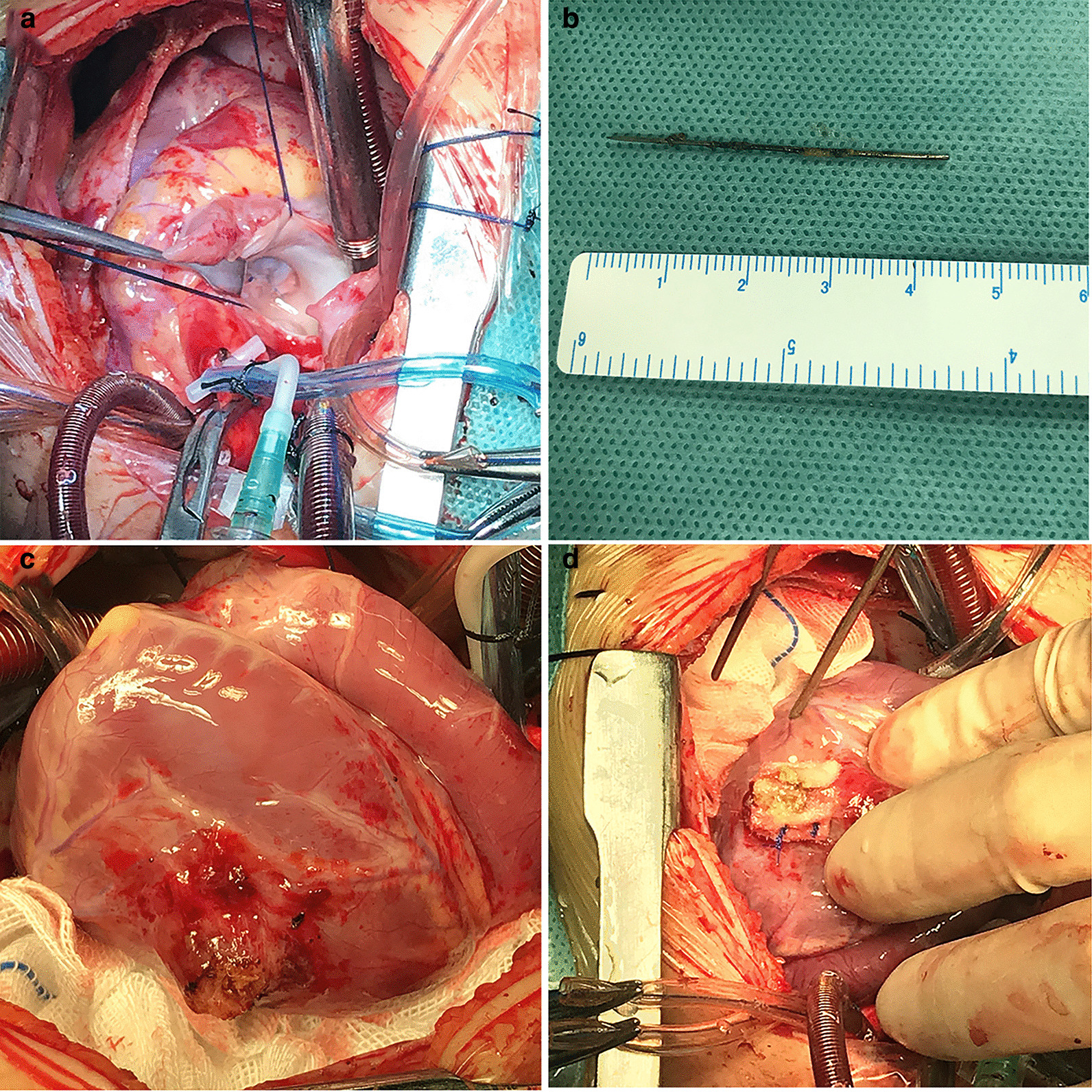
Imaging of the operation. **A** mitral valve was repaired by double-orifice (edge to edge) technique, and no regurgitation. **B** the needle removed from the heart. **C** Adhesion of left ventricular and pericardium. **D** Close crevasse with felt piece

A two-year-old girl was brought to the emergency room because of shortness of breath and chest distress (History of present illness seen Table 1). However, the hemodynamics including heart rate and blood pressure were stable. She also had an intermittent fever for the past one month, and the highest was 39 °C. A 3/6 grade systolic murmur was heard at the apex auscultation area. ECG was nearly normal. Chest X-ray and thoracic CT showed a needle inserted from the chest wall into the heart cavity (Figs. 1 and 2). Echocardiography showed severe mitral valve prolapse and regurgitation (Fig. 3A, Additional file 1: Video 1); the needle was inserted into the left ventricle and papillary muscle (Fig. 3B, Additional file 2: Video 2). We asked the patient’s parents about the medical history of the child; however, they were unaware of how the needle was penetrated the girl’s body. After the exclusion of surgical contraindications, we decided to perform this operation. After thoracotomy, we found that the left ventricular surface had severe hyperplasia and adhesion to the pericardium. However, the needle was invisible. We then established a cardiopulmonary bypass (CPB). After cardiac arrest, we observed that the apex of the needle penetrated from the right atrium-atrial septum into the posterior wall of the left ventricle through the anterior papillary muscle. Anterior papillary muscle and chordae tendineae were partially destroyed and showed infectious endocarditis-associated changes. The mitral valve was severely refluxed. We took the needle out from the mitral orifice (Fig. 4B) and fixed the chordae tendineae to the papillary muscle. However, some chordae tendineae and papillary muscles were destroyed, and moderate-to-severe mitral valve regurgitation persisted. Accordingly, we repaired the mitral valve by double-orifice (edge-to-edge) technique (Fig. 4A). Intraoperative transesophageal echocardiography (TEE) showed mild mitral valve regurgitation (Additional file 3: Video 3). Finally, we separated the left ventricular surface from the pericardium and closed the crevasse by a mattress suture using a felt pledget (Fig. 4C, D). Finally, the girl recovered and was discharged 26 days after the operation. The child was already treated with antibiotics before coming to the hospital, and preoperative blood cultures were negative. However, endocarditis could be diagnosed by 1 major criteria (imaging positive for IE) and 3 minor criteria (predisposition, fever defined as temperature > 38 °C and vascular phenomena) according to 2015 ESC Guidelines for the management of infective endocarditis. As a result, diuretics were used within 2 months after the operation, and antibiotics were used because of endocarditis according to the ESC guidelines of infective endocarditis. Fortunately, she was well at the 2-year follow-up. Echocardiography at the local hospital showed that mitral valve was normal, but she did not come to our hospital for echocardiography examination due to the long distance and economic reasons. Nonetheless, close attention must be paid to the fragile mitral valve as the girl grows. There have been previous reports of a heart stuck by a needle; however, reports on the damage of the papillary muscles and chordae tendineae are rare.

**Table 1:** The time line table of the girl’s symptoms and sign

Time line	Symptoms and sign
Uncertain time	The needle was penetrated the girl’s body
One month	Intermittent fever
2 h	Sudden shortness of breath and chest distress
Present	Come to the emergency room

## Discussion and conclusions

Some cases of cardiac foreign bodies have been reported previously. There are various types of foreign bodies such as needles [5], bullets [6], a fish thorn [7], and a catheter [8] among others. The foreign bodies may penetrate different parts of the heart, and the most commonly reported part is the right ventricle which is at the anterior of the heart. Other parts include the left ventricle, left atrium, right atrium, and pulmonary artery. Whether the cardiac foreign bodies need to be removed through operation depends on the symptoms and possible complications.

The method of surgery for removing metal foreign bodies from the heart is difficult and time intensive but very crucial. With the development of medical equipment and technological advancements, ultrasound has become the main basis for clinically determining the location of foreign bodies [9]. It can determine the location of foreign bodies with an accuracy that traditional X-ray and high-resolution CT cannot achieve. Foreign bodies in the heart are different from foreign bodies in other organs, and once the diagnosis is made, require surgical treatment as soon as possible to remove the foreign bodies to avoid complications such as infection, bleeding, and embolism [10]. In patients with pericardial effusion, irrespective of whether pericardial tamponade occurs, prompt treatment should be conducted as soon as possible to avoid hemodynamic disorders, which can lead to the death of the patient. Once a pericardial tamponade is found, emergency thoracotomy should be performed quickly and decisively. Further, even for those without signs of cardiac tamponade, the foreign body should be removed by early surgery to avoid secondary symptoms. In our case, ultrasound, X-ray, and high-resolution CT were all used. There was no pericardial tamponade. However, ultrasound determined that the needle was in the heart cavity and had destroyed the mitral valve with severe mitral valve regurgitation. The girl showed symptoms of left-sided heart failure.

The surgical approach for the removal of the foreign bodies is individualized based on the types of foreign bodies [9]. It is mostly influenced by the location of the foreign bodies in the heart and the associated injuries caused by foreign bodies. When the foreign bodies are inserted into the cavity of the heart and influence important structures such as the papillary muscle or valve, and especially if endocarditis is observed, CPB is needed. Our case was very rare, wherein the needle penetrated from the intercostal space to the left ventricle and then inserted into the papillary muscle simultaneously. The girl had a fever, shortness of breath, and chest distress because of mitral valve regurgitation and endocarditis. We could not see the head of the needle from the outside of the heart after thoracotomy, and CPB was therefore necessary. With the help of CPB, we could remove the needle from inside the heart cavity. We then successfully repaired the mitral valve, and the girl’s symptoms disappeared.

## Conclusion

Cardiac foreign bodies, especially a needle, are very rare, and ultrasound is an important inspection method. The approaches to handle these are different depending on the associated injuries.

## Supplementary Information

**Additional file 1**. Preoperative Echocardiography showed severe mitral valve prolapsed and regurgitation.

**Additional file 2**. Preoperative Echocardiography showed the needle in the heart cavity.

**Additional file 3**. Intraoperative Transesophageal Echocardiography (TEE) showed mild mitral valve regurgitation, and no mitral valve stenosis.

## Data Availability

Not applicable.

## Abbreviations

CPB: Cardiopulmonary bypass
TEE: Transesophageal echocardiography
